# An IoT System for Remote Health Monitoring in Elderly Adults through a Wearable Device and Mobile Application

**DOI:** 10.3390/geriatrics4020034

**Published:** 2019-05-07

**Authors:** Luis A. Durán-Vega, Pedro C. Santana-Mancilla, Raymundo Buenrostro-Mariscal, Juan Contreras-Castillo, Luis E. Anido-Rifón, Miguel A. García-Ruiz, Osval A. Montesinos-López, Fermín Estrada-González

**Affiliations:** 1 School of Telematics, University of Colima, 28040 Colima, Mexico; lduran1@ucol.mx (L.A.D.-V.); raymundo@ucol.mx (R.B.-M.); juancont@ucol.mx (J.C.-C.); oamontes1@ucol.mx (O.A.M.-L.); fermin_estrada@ucol.mx (F.E.-G.); 2 School of Telecommunications Engineering, University of Vigo, 36310 Vigo, Spain; lanido@gist.uvigo.es; 3 Department of Mathematics and Computer Science, Algoma University, Sault Ste. Marie, ON P6A 2G4, Canada; miguel.garcia@algomau.ca

**Keywords:** older adults, nursing homes, Internet of Things, wearable computing

## Abstract

With the increase in global life expectancy and the advance of technology, the creation of age-friendly environments is a priority in the design of new products for elderly people healthcare. This paper presents a proposal for a real-time health monitoring system of older adults living in geriatric residences. This system was developed to help caregivers to have a better control in monitoring the health of their patients and have closer communication with their patients’ family members. To validate the feasibility and effectiveness of this proposal, a prototype was built, using a biometric bracelet connected to a mobile application, which allows real-time visualization of all the information generated by the sensors (heart rate, body temperature, and blood oxygenation) in the bracelet. Using these data, caregivers can make decisions about the health status of their patients. The evaluation found that the users perceived the system to be easy to learn and use, providing initial evidence that our proposal could improve the quality of the adult’s healthcare.

## 1. Introduction

In recent years, the number of scenarios in which we use the Internet has been increasing, evolving from being static to being social, transactional, and mobile [1]. However, the Internet continues its evolution, and now we talk about connecting “things” (objects/devices) to this network which were not previously designed to have this connectivity but are currently communicating with each other. This evolution has created its own concept: The Internet of Things (IoT), defined as the interconnection to the Internet of any daily use device (or among them) anywhere and anytime, ranging from cell phones, coffee machines, washing machines, and clocks to machine components, such as the engine of an airplane [2]. These objects/things are called nodes, which must operate autonomously and have the ability to transmit/receive small amounts of information, access resources in the cloud, and in some cases, make decisions according to the sensed data [3].

Currently, there are devices with sensors of different types (e.g., movement, environmental, contact) that can be interconnected, through the Internet or other network technology, to be monitored and controlled by a central IoT server [4]. An estimated 21 billion devices on the planet were connected or will connect to the Internet in 2018, representing almost twice the number of the human population, and this number is projected to grow to more than double, 50 billion devices, for 2022 [5].

Furthermore, mobile devices such as smartphones and tablets play a very important role in our daily lives, since they are used in many people’s daily activities (e.g., telephony, phonebook systems, instant messaging). Their technological development has advanced in such a way that for some years, they have been equipped with similar or even greater capabilities than some desktop computing equipment [6].

In light of all of the foregoing, the world of healthcare has acquired concepts such as: eHealth, which is the intersection of medical information, public health and business, regarding services and information provided or improved through the Internet and related technologies [7], and mHealth, which is a derivation of eHealth and consists of the practice of medicine and public health at a distance, supported by mobile devices. These concepts marked the beginning of a revolution in healthcare, which has improved both healthcare and remote patient care through technology.

IoT technologies have penetrated several fields of healthcare, from follow-up to rehabilitation therapies [8], personal monitoring of daily activities [9], reminders of medical appointments and medication intakes [10], to the remote monitoring of vital signs of patients [11]. Recently, advances in sensors and mobile devices have led to the development of wearable devices (wearables) that connect with smart phones to analyze the data obtained from people who use them to monitor their health, give suggestions to improve it, and even predict hidden diseases through intelligent algorithms applied to the data sensed from devices such as: Bracelets, watches, lenses, gloves, and even implants in the patients’ bodies [12]. Through medical IoT technology, physicians or caregivers responsible for the health of patients can remotely know, in real time, the physical condition of people.

An important sector that has benefited from eHealth are the elderly, who can live in age-friendly environments and at the same time take care of their health using technology. The main types of housing in this sector are: Independent (e.g., own home), private housing with assistance, and nursing homes [13]. Nursing homes for older adults are facilities with medical and care staff available to look after the health of the patients who live there, who are regularly monitored for vital signs, spatial tracking, and health in general. This is an ideal environment to implement IoT technology with the support of wearables to improve the process of monitoring, supervision, and decision making regarding the health of the residents.

This study describes the design, development, and usability assessment of Abuelómetro, a technological platform created by the authors, whose objective is focused on providing a support tool to the personnel responsible for the care of elderly people in nursing homes, in order to monitor, in real time, the health status of their patients and keep in constant communication with family members, through the IoT and a wearable device.

The rest of the paper is organized as follows: Section 2 focuses on related works pertinent to the research; materials and methods are shown in Section 3; in Section 4, the results and discussion are presented; and finally, Section 5 presents the conclusions.

## 2. Related Works

This section discusses some alternative approaches that have been proposed in the context of healthcare for the elderly through remote monitoring.

### 2.1. Mobile Monitoring to Predict Medical Conditions

This project uses wearable technology, watches and phones that have the Android operating system. The objective is to collect and process data from sensors placed in patients with medical conditions to detect or, ideally, predict episodes such as chronic obstructive pulmonary disease (COPD), seasonal affective disorder (SAD), and bipolar affective disorder (BAD). The smartphone requires users to complete a questionnaire that describes their health once per day. After finishing the questionnaire, patients are asked to blow into the microphone as a measure of their maximum expiratory flow. The data collected during the day are sent to a remote server to be analyzed [14]. When evaluated with patients, preliminary results indicated that they are willing to use this wearable device.

### 2.2. LifeShirt

The LifeShirt ambulatory monitoring system consists of a chest and shoulder strap that records cardiorespiratory measurements in patients with medical or psychological disorders in an outside a laboratory environment. Its sensors measure: Respiratory rate, heart rate, posture, and skin temperature [15]. This device has been tested, and the results suggest that it provides a reliable way to monitor patients who use it [16].

### 2.3. Vita-Data

Vita-Data is an innovation that combines hardware and software to help to monitor vital signs and send alerts in real time to the patient’s caregivers of children, seniors, and people with disabilities. It has sensors that measure body temperature, heart rate, and blood oxygenation, in real time, while comparing the readings of the sensors with the parameters defined by the doctor, and in cases where it detects an anomaly, it sends an alert to another bracelet that the caregiver uses [17].

The three analyzed works use very similar sensors that monitor vital signs, but the technologies are different; although they are wearable technologies, the implementations have been in the form of smart watches, bracelets and belts, each of them with the purpose of adapting to the type of patient who will use it, as well as the physical activity that he performs.

Our goal is to create a system that provides specific support for caregivers of elderly people living in nursing homes.

This implies the need to carry out an in-depth study of this scenario in order to obtain knowledge about the problems and needs that are faced every day.

## 3. Materials and Methods

This research uses the user-centered design as a methodology, which aims to know and understand the user’s needs, limitations, behavior, and characteristics, in order to ensure that the final product is of quality and will be accepted by the end user.

### 3.1. Initial Understanding

In order to accomplish the main objective and achieve the initial understanding of the users, five residences dedicated to the care of the elderly were contacted in the State of Colima in Mexico. A total of 14 caregivers of these adults were interviewed. For this purpose, we developed a semi-structured interview with topics relevant to the research and standards and recommendations for qualitative interviews were followed [18].

The interviews were analyzed through a comparative verification of the evidence that resulted in the identification of key points that should be addressed in this project. Below, some of the most relevant findings are explained.

It was found that all caregivers keep a daily record of the health status of all elderly people in the residence, being the most important document for the health control of patients. In this registry, which is usually on paper, the clinical follow-up of the elderly person is reflected, which contains physician prescriptions or in some cases instructions issued by the elderly’s relatives. It also includes data such as blood pressure and glucose level, and in some cases the time interval in which these values will be measured, either daily, every other day or every week.

The main communication mode between caregivers and family members is the telephone, followed by messaging systems such as WhatsApp and Facebook Messenger. This communication is mainly about updates of health status and the needs of the elderly, for example, lack of medicines, external medical attention, or to invite them to events such as Mother’s or Father’s Day.

When an older adult enters a nursing home, it is necessary to name a direct person responsible, who must be in constant communication with the residence and the elderly. In addition, the relative must provide all the medical information of the older adult.

In residences with medical staff, an initial assessment is performed to identify the medical state in which the elderly person is admitted. Depending on the geriatric residence, this diagnostic includes the measurement of body temperature, heart rate, oxygen saturation, blood glucose level, and blood pressure; additionally, it was found that anthropometric variables such as waist circumference, the patient’s height, and body weight are taken into account in a nursing home.

A condition is that the admitted adults are not violent, and as part of their registration information, their anthropometric data is recorded; if necessary, laboratory tests are performed to corroborate the health of the elderly in their admittance and to initiate a clinical history and a medical record of the patient. Some of the main problems detected in the residences are:

1. Many older adults, with few caregivers to care for them;
2. Little communication between the caregiver and the family;
3. Health monitoring is registered mainly on paper;
4. Very low involvement of the family members in the care of the elderly.

Based on the analysis of this information, the characteristics that the system of Abuelómetro should cover to support the healthcare of the elderly living in nursing homes were obtained.

### 3.2. Envisioned System

We envisioned that to improve the healthcare of seniors living in nursing homes, the system needs to address the following aspects:

#### 3.2.1. Medical Record

The system must be able to replace the traditional paper log, storing the records of the elderly in an electronic database and offer access to them in a simple and immediate way, with data updated in real time. In this way, physicians and caregivers can register daily life activities, food, medicines, health conditions, injuries, medical treatments, photos, and notes.

#### 3.2.2. Communication with Family

One of the main problems that was found is the lack of involvement of the family. To lessen this, a function was included to send reports to the family with details of the daily life of the patient: Participation in activities, illnesses, therapies, food, and medication intakes. Likewise, the family can give an answer and instructions to the caregiver through this function, to speed up the attention and get involved in the decision making about the care of the elderly.

#### 3.2.3. Remote Monitoring

Through the wearable IoT device, Abuelómetro will have remote monitoring of the vital variables of the adult through the Internet in real time; the variables to be collected by the sensors were selected based on the user’s interviews and whether they can be measured without intrusive sensors and human intervention: Heart rate, body temperature, and blood oxygenation. These data can be visualized by the doctors, caregivers and relatives of the patient in real time within the mobile application.

#### 3.2.4. Medical History

When the older adult is admitted into the residence, an initial clinical history should be created, with data such as surgical history, obstetric history, prescriptions, allergies, social data (religion, occupation, studies), habits, and vaccines. Additionally, through this functionality, the health of the elderly can be monitored through a digital registry.

#### 3.2.5. Alert Notifications

When the IoT system detects a possible alarm situation with the patient’s health, it must be able to generate an alert for caregivers and family members.

### 3.3. System Architecture

**Figure 1:**
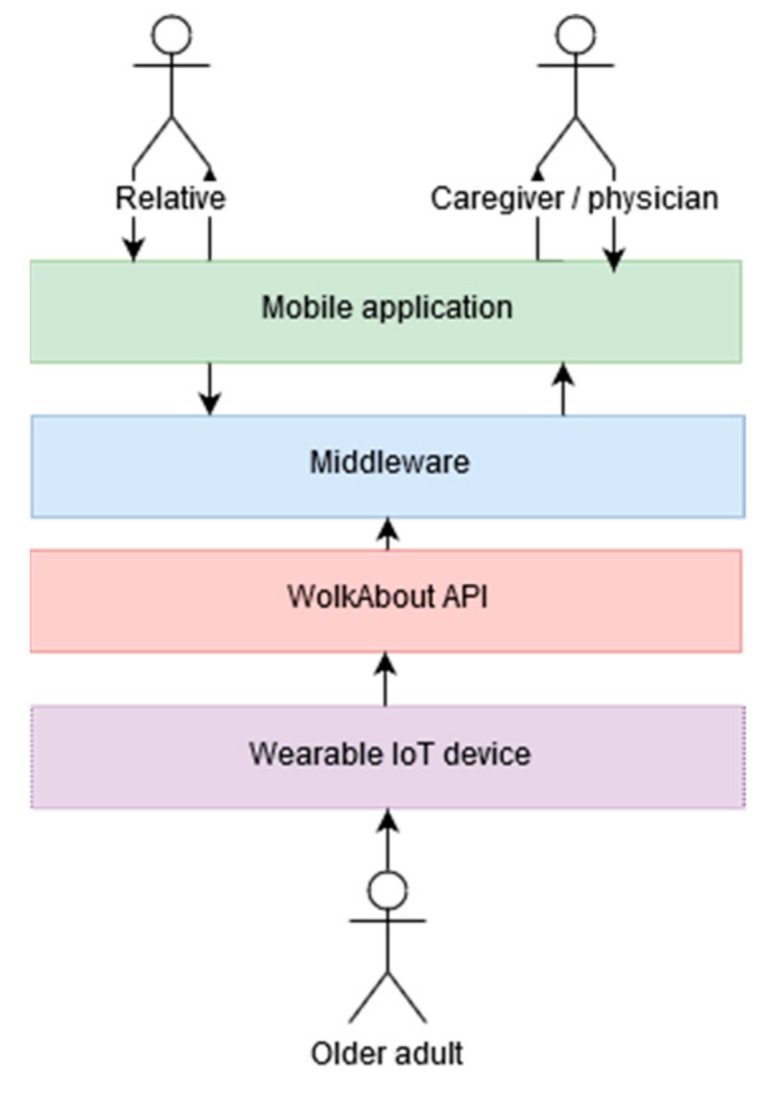
System architecture of Abuelómetro.

As illustrated in Figure 1, the system architecture of Abuelómetro consists of several layers.

#### 3.3.1. Wearable IoT device

**Figure 2:**
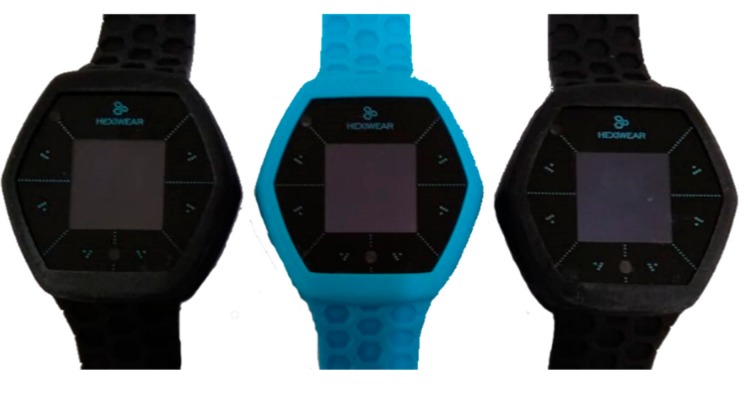
Hexiwear biometric bracelets.

The first layer consists of the wearable IoT device, which corresponds to the Hexiwear biometric bracelet. It is a device with capabilities to become an IoT node and includes sensors to measure heart rate, body temperature, and blood oxygenation [19], In addition, it is energy-efficient and has wireless communication and a touch screen. It was selected because both software and hardware are open-source, so it is fully configurable to the needs of each project (see Figure 2).

#### 3.3.2. WolkAbout API

The Hexiwear bracelet is supported by a platform called “WolkAbout IoT Platform”, among the components of which is a mobile application for smartphones and which stores all the information generated by the bracelet sensors in the cloud. In this way, data such as oxygenation, heart rate, and temperature are made available to developers through its application programming interface (API), in such a way that applications can be developed to recover the information that is stored in their servers [20].

#### 3.3.3. Middleware

For the API to communicate with the patient data and the application, it was necessary to develop a middleware layer. This middleware was implemented using the service-oriented computing (SOC) paradigm [21], which is based on services that expose their functionality through a web API. This API provides access to the data of the users wearing the IoT device, which are stored in the cloud and are available to any application through the programming code.

**Figure 3:**
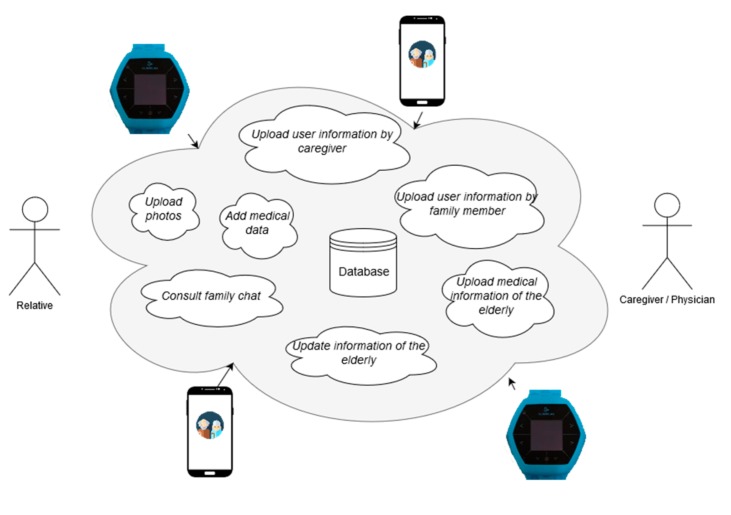
Services available in the cloud and accessible through the middleware. *Upload photos*: Caregiver-type users can share photos with family-type users. *Upload user information by caregiver*: This service allows uploading and updating information related to caregiver-type users. *Add medical data*: The service allows caregiver-type users to create new classifications to personalize the medical data of the elderly at their care. *Upload user information by family member*: This service allows uploading and updating information related to family-type users. *Consult family chat*: Caregiver-type users can access the conversation history with the relatives of the elderly at their care. *Update information of the elderly*: This service allows uploading and updating personal information of the elderly. *Upload medical information of the elderly*: The functionality of this service is to allow caregiver-type users to upload medical data of the elderly who are in their care.

In Figure 3, the services provided by the middleware are shown, and they are described below.

#### 3.3.4. Mobile Application

This layer is the subsystem that allows family members and caregivers to interact with the monitoring of patients’ vital signs and their medical information.

### 3.4. Development

The Abuelómetro system was developed using React Native. The device used for development and testing was a Samsung Galaxy S7 (Samsung Electronics, Suwon, South Korea) this smartphone includes an Android operating system Nougat 7.0 version. For storage information in the middleware, MongoDB was used, which is a tool that allows us to develop databases oriented to documents instead of relations (NoSQL database). To implement the middleware, we studied the implications of performance for a real-time web API; our first option was the PHP/Apache stack, but after analyzing the literature, we found that Node.js technology outperformed the main web development technologies (PHP/Apache, PHP/Nginx) in computational performance [22,23] and have been used by real-time high-speed and scalable web applications [24,25,26]; for the abovementioned, the middleware was developed with the Node.js technology.

Mobile computing has several advantages in healthcare, but at the same time, privacy and security are essential for any health monitoring technology [27]; many challenges arise because the privacy of patients can be compromised and the quality of the data for medical use can be leaked if a cyber-attack occurs. At this stage of development, no additional security measures have been implemented to prevent the leakage of patient information; this is considered in the next iteration.

### 3.5. Evaluation

An assessment of user experience of the system was performed. In the evaluation, the systems usability scale (SUS) was used [28]; this is a very useful instrument to evaluate the user’s perception in a simple and reliable way (see Appendix A).

It is acceptable to evaluate with five users to detect the majority of problems with usability [29]. To fulfill this criterion, five users were selected to perform usability testing. The sample was selected by convenience sampling, per the availability of the subjects; gender distribution was: 80% female and 20% male. The information of the users is shown in Table 1.

#### Process

First, the system prototype was explained to the users. Next, users were given a demonstration of the mobile application. Later, a list of tasks was assigned to be performed in the application. Finally, participants were asked to fill out the SUS questionnaire, which is a 10-item Likert scale instrument to assess the platform usability.

## 4. Results and Discussion

### 4.1. Development

A fully functional prototype of the mobile application Abuelómetro and the middleware to interface with the API was developed to communicate with the WolkAbout IoT Platform.

**Figure 4:**
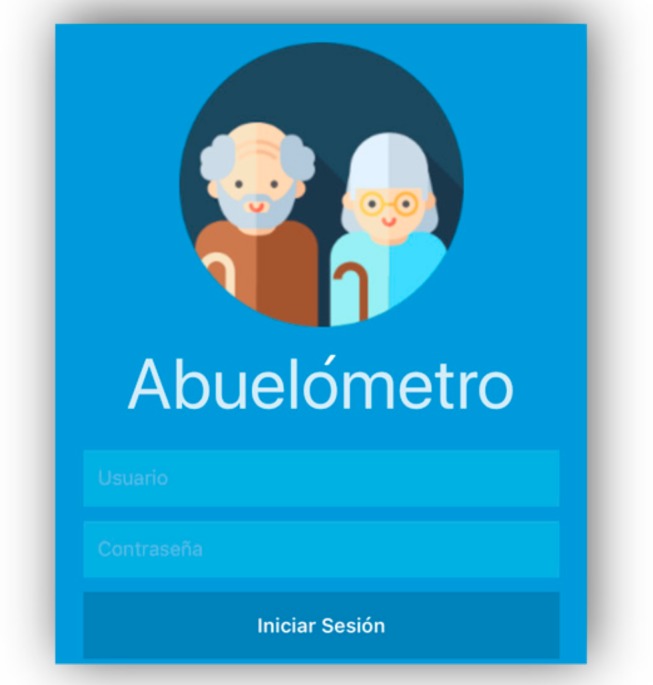
Login screen of Abuelómetro.

The visualization of the data can be seen in graphical form by caregivers and relatives of the elderly; it is necessary to install the mobile application on their smartphones and have an Internet access service. Figure 4 shows the authentication interface in the application Abuelómetro.

All the envisioned characteristics of the system were completed in the Abuelómetro prototype. Below, the main interfaces of each of these characteristics are shown.

#### 4.1.1. Medical Record

**Figure 5:**
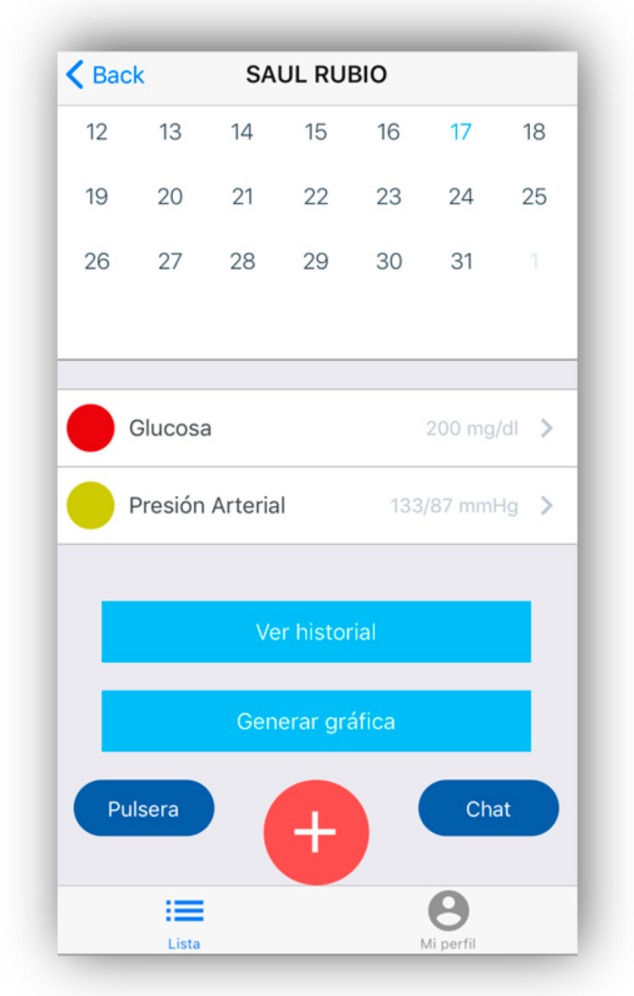
Medical record.

The caregiver-type user can access and view the historical data on a specific date in the calendar. In addition, depending on the selected day, you can see the history of the previous days (see Figure 5).

#### 4.1.2. Communication with Family

**Figure 6:**
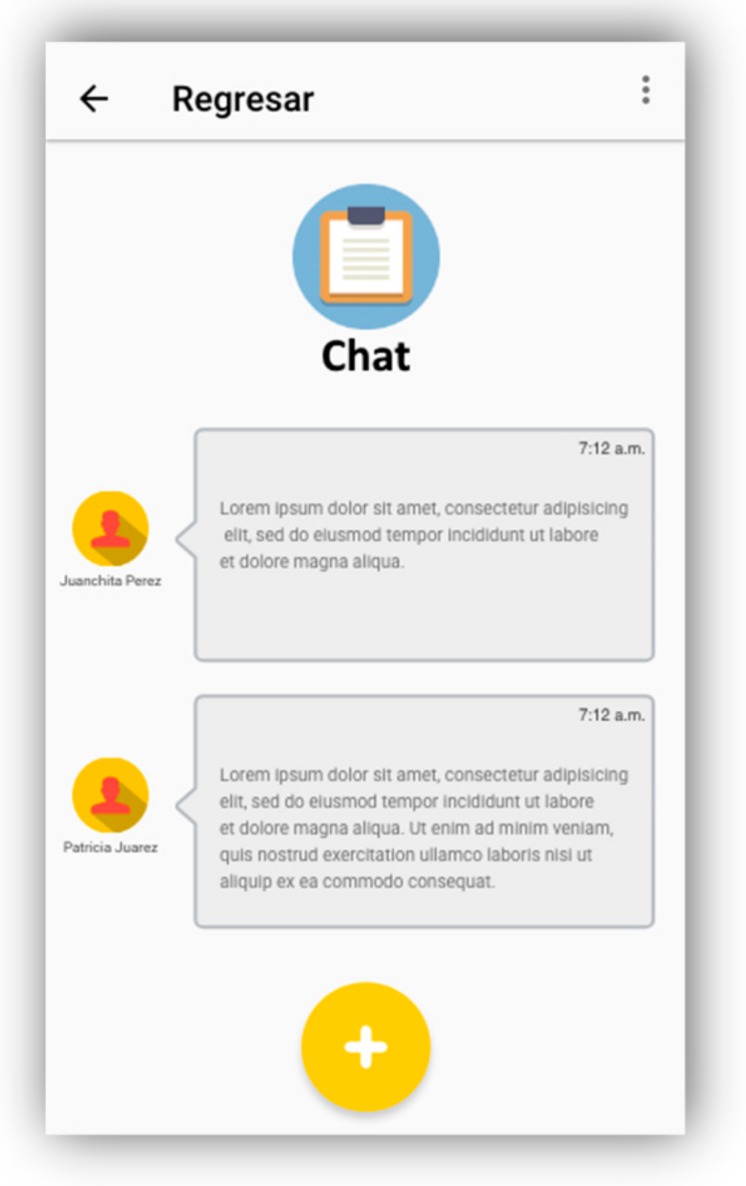
Chat to communicate with family.

An instant messaging module that allows direct communication between caregivers and family members was developed; this will help to avoid having to use different applications to track the health control and the communication among family members and caregivers (see Figure 6).

#### 4.1.3. Remote Monitoring

**Figure 7:**
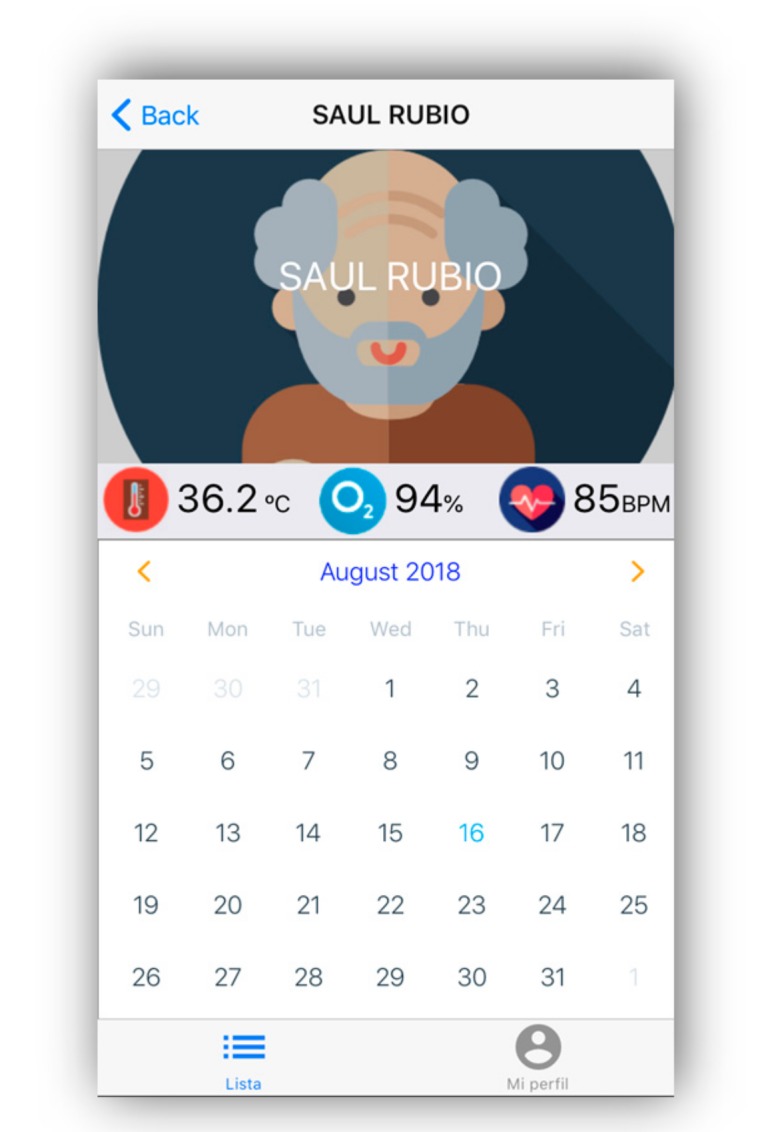
Real-time readings of the device’s sensors.

The caregiver-type user will be able to access the main screen of a senior adult, in which they will be able to visualize the information generated in real time by the sensors through the biometric bracelet. In addition, they will be able to navigate the information of different days in order to visualize historical data (see Figure 7).

#### 4.1.4. Medical History

**Figure 8:**
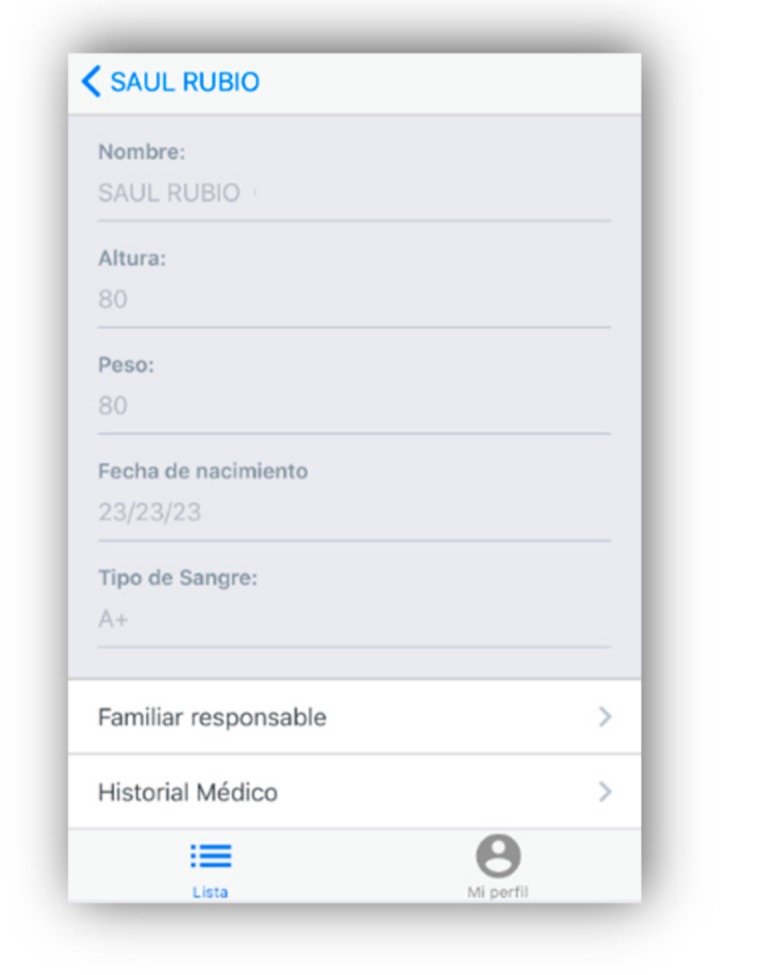
Clinical history of the elderly.

Caregiver-type users can access and view the medical and personal data of the elderly who are at their care, as can be seen in Figure 8.

#### 4.1.5. Alert Notifications

**Figure 9:**
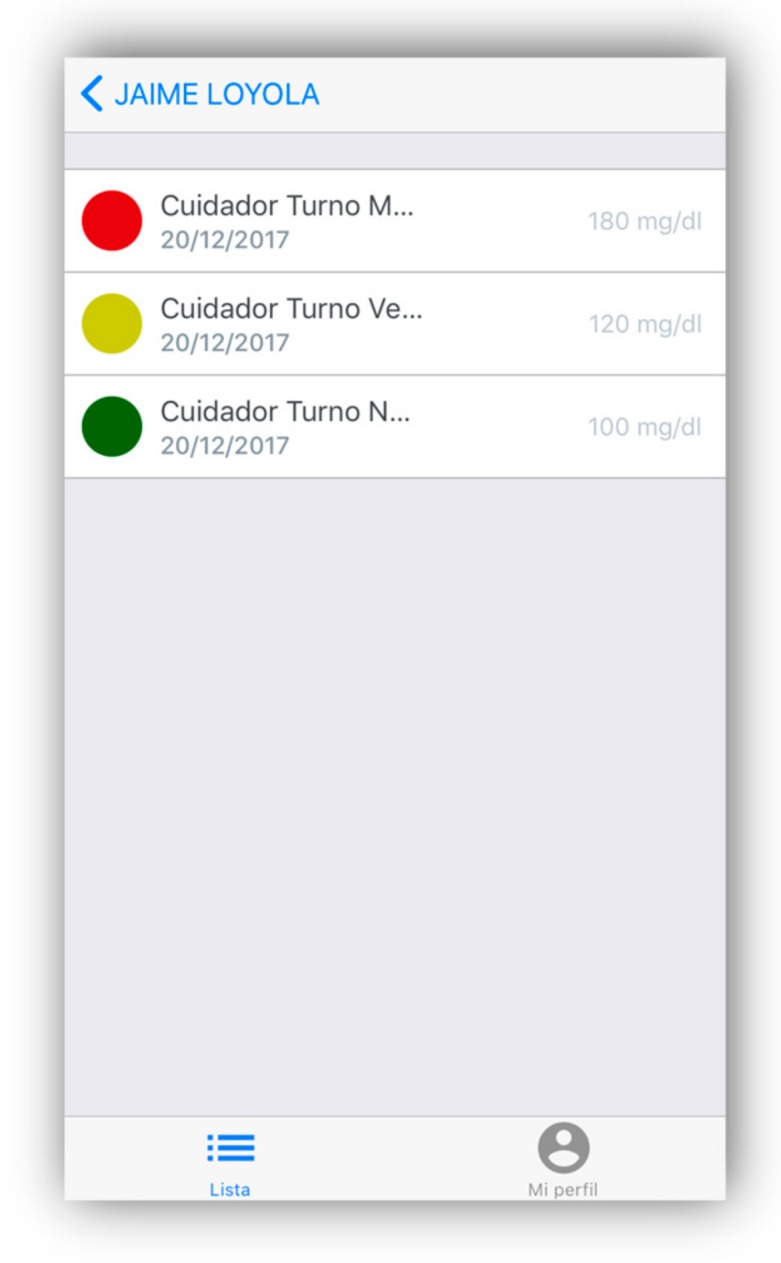
Alerts with color codes according the sensed data by the bracelet.

Abuelómetro has algorithms that analyze the sensed data obtained by the sensors in real time; if it detects that the sensed data are outside normal parameters, the display will indicate the alert by color codes, similar to those used in traffic lights (see Figure 9). Additionally, an alert message will be sent to the messaging application, both to family members and caregivers. The algorithms to analyze the sensed data are based on deriving secondary context specifying a set of rules to infer it from the data collected by the sensors.

### 4.2. Usability

The purpose of the SUS questionnaire is to provide an instrument to measure usability that is easy to complete and evaluate.

Once the user completes the 10 items, the questionnaire is scored as follows:

1. In the odd items, subtract one from the position marked by the user.
2. For even items, subtract the position marked from five.
3. Add up these new values from responses and multiply that total by 2.5.
4. Then we obtain a general value of usability on a scale of 0 (null usability) to 100 (excellent usability). It is not a percentage.

Table 2 shows the values that each user gave to the questions of the SUS questionnaire, the SUS value for each of the users, as well as the calculated average SUS score of all users.

The SUS evaluation gave a score of 83.0, so according to the theory [30], it is considered with very good usability, because you need to score above 80.3 to get an A; at this point, users are more likely to recommend Abuelómetro to their friends.

**Figure 10:**
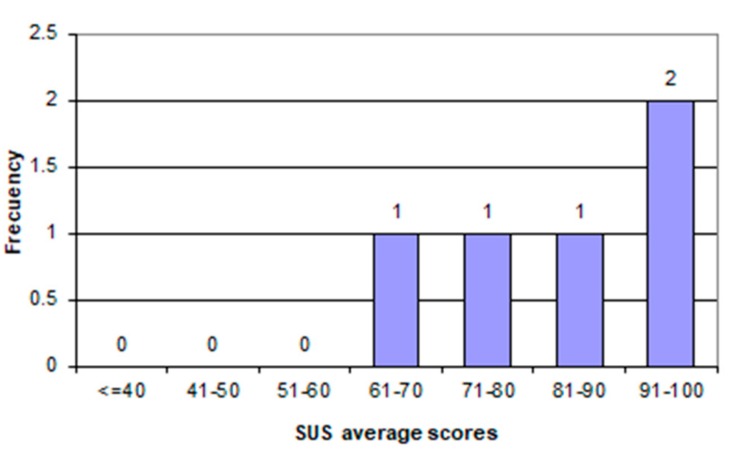
Average SUS scores.

Figure 10 shows the distribution of the frequencies of each participant of the evaluation. As can be seen, the users individually assessed Abuelómetro with a positive perception.

## 5. Conclusions

This paper presents the design and development of an IoT system for the remote monitoring of elderly people living in nursing homes, through a mobile application and a wearable device.

The design was based on a contextual study in geriatric residences, in which semi-structured interviews were applied to the personnel responsible for the care of the elderly.

The development of the prototype showed that it is feasible to carry out and implement the proposal of this research. In addition, it is low-cost and aligned to the IoT paradigm; the most important characteristics are: Real time tracking of the general conditions of the patients, the fact that it allows interaction between caregivers and family, that it is accessible remotely, and that the highest cost is the wearable device, which costs less than $100 US.

The results of the usability evaluation were very promising and positive, showing that Abuelómetro was well received by the users, providing initial evidence that our proposal could improve the quality of the adult’s healthcare, and additionally, it provided valuable information that can be used to correct the usability problems that may affect the acceptance of the technology by end users.

As future work, a long-term evaluation in geriatric residences is planned, to validate directly with potential users the benefit that this system can bring to them when implementing it with their patients.

## Figures and Tables

**Table 1:** Characteristics of the users.

User	Gender	Age
1	Female	34
2	Female	32
3	Female	55
4	Female	28
5	Male	33

**Table 2:** Systems usability scale (SUS) values by users.

User	Q1	Q2	Q3	Q4	Q5	Q6	Q7	Q8	Q9	Q10	SUS Value
1	5	2	5	1	5	2	5	1	5	1	95.0
2	4	2	4	1	5	2	5	4	2	1	75.0
3	5	3	4	3	4	3	5	3	4	3	67.5
4	4	2	4	2	4	1	4	1	4	1	82.5
5	5	2	5	2	5	1	5	1	5	1	95.0
	Average SUS score										**83.0**

## Appendix A

The systems usability scale (SUS) provides a reliable tool for measuring usability [28]. Participants will rank each question from five response options based on how much they agree with the statement they are reading; from Strongly agree to Strongly disagree. Next, the SUS questionnaire is presented:

1. I think that I would like to use this system frequently.
2. I found the system unnecessarily complex.
3. I thought the system was easy to use.
4. I think that I would need the support of a technical person to be able to use this system.
5. I found the various functions in this system were well integrated.
6. I thought there was too much inconsistency in this system.
7. I would imagine that most people would learn to use this system very quickly.
8. I found the system very cumbersome to use.
9. I felt very confident using the system.
10. I needed to learn a lot of things before I could get going with this system.
